# Overexpression of *S*-Adenosyl-l-Methionine Synthetase 2 from Sugar Beet M14 Increased *Arabidopsis* Tolerance to Salt and Oxidative Stress

**DOI:** 10.3390/ijms18040847

**Published:** 2017-04-18

**Authors:** Chunquan Ma, Yuguang Wang, Dan Gu, Jingdong Nan, Sixue Chen, Haiying Li

**Affiliations:** 1 Key Laboratory of Molecular Biology, College of Heilongjiang Province, College of Life Sciences, Heilongjiang University, Harbin 150080, China; chqm0913@163.com (C.M.); gudan9865@sina.com (D.G.); jingdongnan09@163.com (J.N.); 2 Engineering Research Center of Agricultural Microbiology Technology, Ministry of Education, Heilongjiang University, Harbin 150080, China; 3 Key Laboratory of Sugar Beet Genetic Breeding of Heilongjiang Province, Heilongjiang University, Harbin 150080, China; wangyuguang0920@hotmail.com; 4 Department of Biology, Genetics Institute, Plant Molecular and Cellular Biology Program, University of Florida, Gainesville, FL 32610, USA

**Keywords:** sugar beet M14, *S*-adenosyl-l-methionine synthetase, salt stress, polyamine, antioxidant system

## Abstract

The sugar beet monosomic addition line M14 is a unique germplasm that contains genetic materials from *Beta vulgaris* L. and *Beta corolliflora* Zoss, and shows tolerance to salt stress. Our study focuses on exploring the molecular mechanism of the salt tolerance of the sugar beet M14. In order to identify differentially expressed genes in M14 under salt stress, a subtractive cDNA library was generated by suppression subtractive hybridization (SSH). A total of 36 unique sequences were identified in the library and their putative functions were analyzed. One of the genes, *S*-adenosylmethionine synthetase (*SAMS*), is the key enzyme involved in the biosynthesis of *S*-adenosylmethionine (SAM), a precursor of polyamines. To determine the potential role of *SAMS* in salt tolerance, we isolated *BvM14-SAMS2* from the salt-tolerant sugar beet M14. The expression of *BvM14-SAMS2* in leaves and roots was greatly induced by salt stress. Overexpression of *BvM14-SAMS2* in *Arabidopsis* resulted in enhanced salt and H_2_O_2_ tolerance. Furthermore, we obtained a knock-down T-DNA insertion mutant of *AtSAMS3*, which shares the highest homology with *BvM14-SAMS2*. Interestingly, the mutant *atsam3* showed sensitivity to salt and H_2_O_2_ stress. We also found that the antioxidant system and polyamine metabolism play an important role in salt and H_2_O_2_ tolerance in the *BvM14-SAMS2*-overexpressed plants. To our knowledge, the function of the sugar beet SAMS has not been reported before. Our results have provided new insights into SAMS functions in sugar beet.

## 1. Introduction

Soil salinity is a serious ecological problem that affects crop distribution and yield around the world [1,2,3]. More than 6% of land throughout the globe is affected by salinization [4]. Thus, improving the salt tolerance of crops to utilize saline soil is of high urgency [5,6]. High concentrations of salt usually lead to ionic imbalance, oxidative damage and nutrient deficiency in plants [2]. In order to adapt to the saline environment, plants can use some strategies allowing for adaptation, which include efflux of salt ions, compartmentalization of Na^+^ in vacuoles, synthesis of osmolytes, and increased synthesis of antioxidant enzymes [7]. In these adaptation processes, salt stress regulatory genes are induced, leading to changes in the protein levels that enable adaptation to the salinity conditions. For instance, many genes involved in signal transduction and redox reaction have been identified in several plant species [8]. It is reported that about 2300 ESTs (Expressed equence tags) in some halophytes showed differential expression under salt stress [9,10,11]. In addition, numerous proteins have exhibited salt stress responses as identified by proteomics studies in several halophytes [12,13,14,15].

*S*-adenosyl-l-methionine (SAM) synthetase, one of the salt-responsive genes, is an important enzyme in the synthesis of SAM. Usually, SAM synthesized by SAM synthetase (SAMS) from methionine and ATP, forms a universal methyl group donor involved in numerous transmethylation reactions [16]. It plays a vital role in metabolism and development regulation, as well as abiotic and biotic stresses [17,18]. Additionally, it functions as a precursor for the synthesis of polyamines (PAs), which are involved in regulating plant responses to abiotic or biotic stresses [19]. In the process of PA biosynthesis, SAM can be decarboxylated by SAM decarboxylase (SAMDC) to form decarboxylated SAM (dcSAM). Then, dcSAM provides aminopropyl groups to putrescine (Put) for sequential formation of spermidine (Spd) and spermine (Spm), catalyzed by Spd synthase (SPDS) and Spm synthase (SPMS), respectively. Put, Spd and Spm are major constituents of polyamines in plants. It is reported that up-regulation of polyamine synthesis by SAMDC-overexpressing tobacco plants displayed a significant increase in the contents of soluble conjugated PAs and resulted in enhanced tolerance to salinity [20]. On the basis of the SAM functions, a hypothesis of SAMS involved in plants abiotic or biotic stress tolerance was proposed. For example, transgenic plants overexpressing *SlSAMS1* exhibited a strong tolerance to alkali stress and maintained a balance of nutrients under stress conditions [21]. Overexpressing *MfSAMS1* in transgenic plants led to high accumulation of SAM and promoted polyamine synthesis, which in turn improved H_2_O_2_-induced antioxidant protection and increased tolerance to various abiotic stresses [22]. It is also reported that overexpression of *SsSAMS2* from a halophyte plant *Suaeda salsa* in tobacco led to salt stress tolerance [23].

Sugar beet monosomic addition line M14 was acquired from the hybridization between *Beta vulgaris* L. and *Beta corolliflora* Zoss [24]. The M14 line has shown characteristics of apomixis and tolerance to abiotic stress [25,26,27]. In this study, we employed suppression subtractive hybridization (SSH) to investigate alterations in the transcriptional profiles of the M14 line under salt stress. Furthermore, *BvM14-SAMS2* was found to be increased in the transcriptional level under NaCl treatment. In order to determine the role of salt-induced *BvM14-SAMS2* in salt response, *BvM14-SAMS2* was cloned using the rapid amplification of complementary deoxyribonucleic acid ends (RACE) method. To investigate the gene functions, transgenic *Arabidopsis* overexpressing *BvM14-SAMS2* was generated and used to examine antioxidant activity and polyamine contents. The results demonstrated that the overexpression of *BvM14-SAMS2* can confer salt and H_2_O_2_ stress tolerance in the transgenic plants.

## 2. Results

### 2.1. Identification of Differentially Expressed M14 Genes under Salt Stress Using SSH

**Figure 1:**
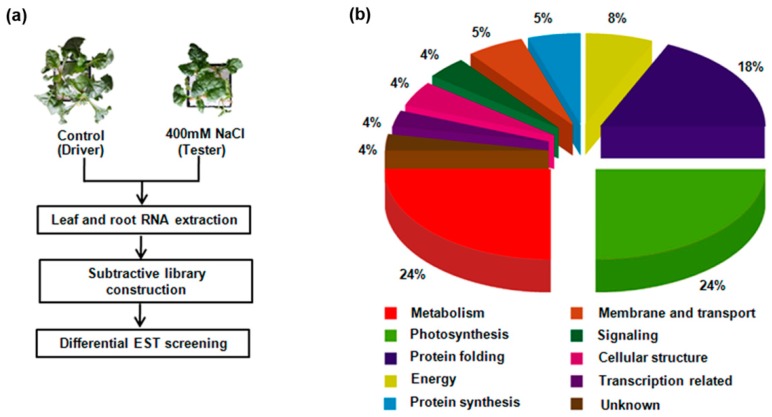
Experimental scheme of screening differentially expressed genes by suppression subtractive hybridization (SSH) and functional classification of the identified genes using the UniProt database. (**a**) Experimental scheme; (**b**) Functional classification of the differential genes; EST stands for expressed sequence tag.

To identify the differentially expressed genes in the M14 line under salt stress, SSH was employed to profile differential gene expression following 400 mM NaCl treatment for 7 days. The cDNAs synthesized from the sugar beet M14 root and leaf mRNAs under control conditions were used as drivers, and those from roots and leaves under salt stress were selected as testers. A total of 500 colonies were randomly selected for sequencing. After counting assembly, 36 unigenes were acquired and annotated by comparison with the non-redundant (Nr) database using Blastx (Table 1). Furthermore, gene classification was carried out by the Gene Ontology (GO) method (Figure 1). For example, many of the genes were classified in terms of catalytic activity (39%) according to their molecular functions. Interestingly, among the differentially expressed genes, leaf (L) 22 and root (R) 6 EST clones showed high homology with plant *SAMS*, which has been reported to play an important role in stress tolerance [17,18].

### 2.2. Cloning of a BvM14-SAMS2 Gene and Sequence Analysis

Based on the EST sequences (L 22 and R 6) matched to a *BvM14-SAMS2* gene, a full-length cDNA clone was obtained using a 5′-/3′-RACE extension method. Sequence analysis confirmed the clone to be the *SAMS* gene. As shown in Supplementary Materials Figure S1, the full-length *BvM14-SAMS2* was comprised of 1538 bp, containing an open reading frame of 1182 bp nucleotides, which encodes a 393-amino acid protein with a molecular mass of 42.99 kDa and a pI (isoelectric point) of 5.59. No signal peptide was found.

Phylogenetic analysis of *BvM14-SAMS2* was performed using a neighbor-joining method with MEGA4.1. The phylogenetic tree reflects both the taxonomy and specificity. As shown in Figure S2, *BvM14-SAMS2* forms a clade with the *Arabidopsis thaliana AtSAMS3*. The result showed that the *BvM14*-*SAMS2* was closely related to monocotyledon and other plants, and *SAMS* genes were relatively conserved in evolution.

### 2.3. Analysis of BvM14-SAMS2 Response to Salt Stress

**Figure 2:**
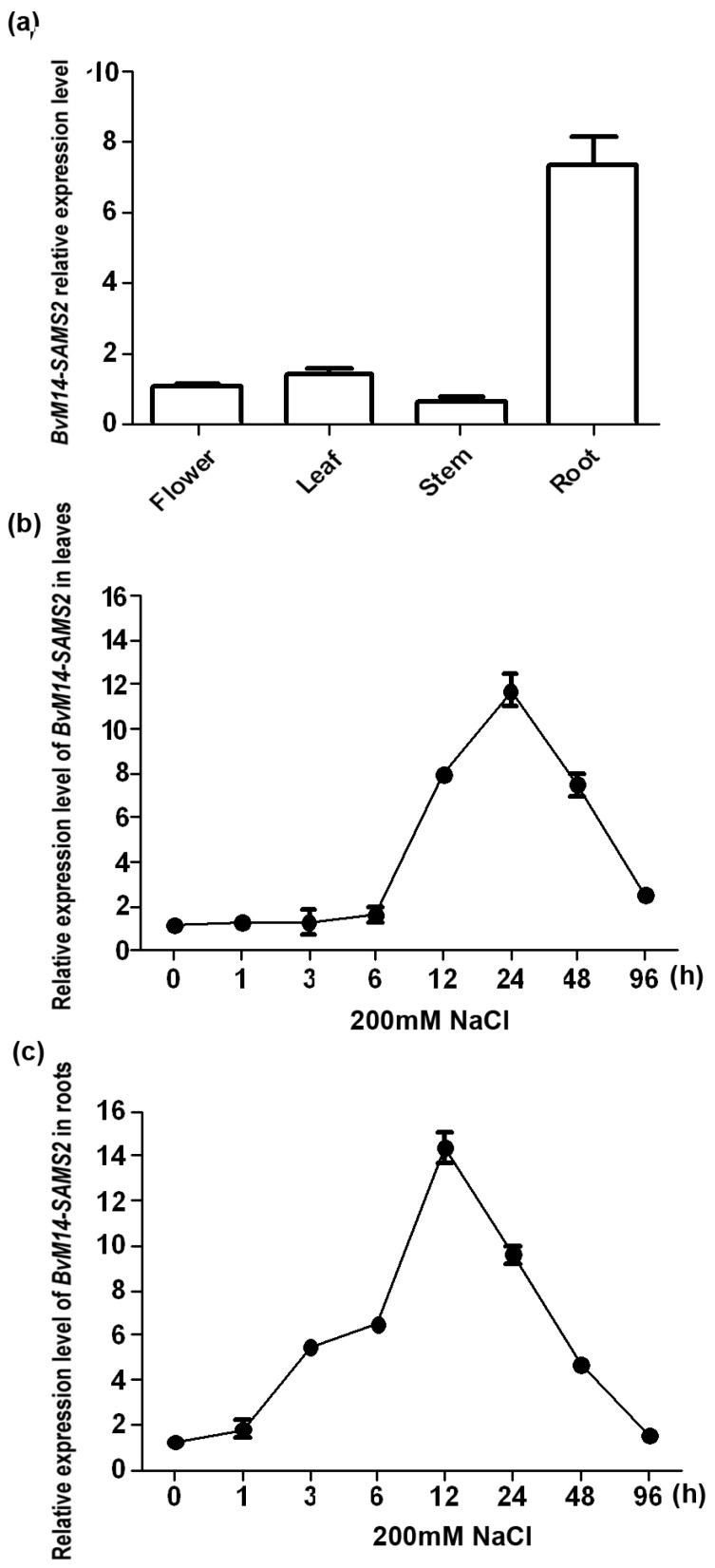
Tissue specific expression of *BvM14-SAMS2* gene in the M14 plants and induction of the *BvM14-SAMS2* mRNA in response to salt stress. (**a**) Real time-PCR detection of *BvM14-SAMS2* gene in different tissues. Time-course analysis of *BvM14-SAMS2* relative expression levels in leaves (**b**); and roots (**c**) of the M14 plants under 200 mM NaCl stress. Data for real time-PCR analysis are the means of three biological replicates and three technology replicates (standard deviation, SD), separately. Each replicate contains five sugar beet seedlings. The 18S rRNA gene was used as the internal control for relative expression analysis.

*BvM14-SAMS2* transcript levels in different tissues were detected. Under normal control conditions, high levels of *BvM14-SAMS2* transcript were found in roots, but a small quantity of *BvM14-SAMS2* transcript was detected in flowers, leaves and stems (Figure 2a). We also determined *AtSAMS3* gene expression in different tissues of *A. thaliana* (Figure S3). *AtSAMS3* expression was strong in roots, and its expression patterns were not substantially different from those of *BvM14-SAMS2* in sugar beet M14. These results confirmed that *BvM14-SAMS2* is closely related to *AtSAMS3*, and we speculated the function of *BvM14-SAMS2* in sugar beet M14 may be similar to *AtSAMS3.*

The responses of *BvM14-SAMS2* mRNA to salt stress were determined in roots and leaves using quantitative reverse transcription-polymerase chain reaction (RT-PCR). The induction of *BvM14-SAMS2* transcript was found much earlier in roots than in leaves (Figure 2b,c). High levels of *BvM14-SAMS2* transcripts were observed at 12 h and 24 h after salt treatment in roots and leaves, respectively (Figure 2b,c). These results showed that the expression of *BvM14-SAMS2* was significantly up-regulated by salt stress.

### 2.4. Overexpression of BvM14-SAMS2 Confers Enhanced Salt and H_2_O_2_ Tolerance in Arabidopsis

**Figure 3:**
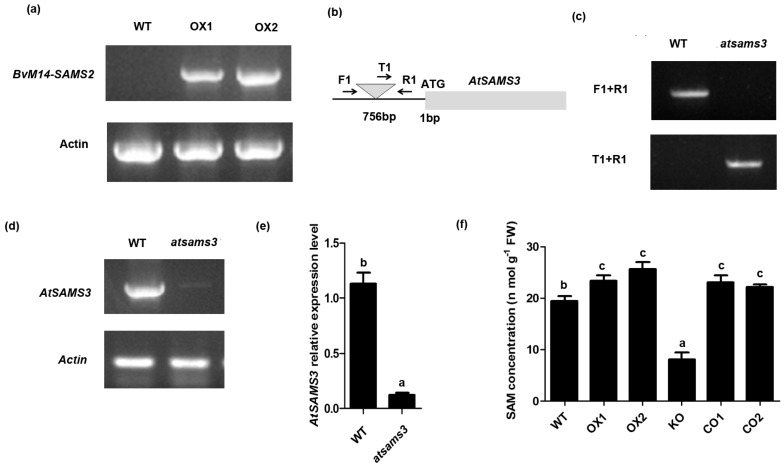
Identification of *atsams3* mutant and overexpressed *BvM14-SAMS2 in Arabidopsis* plants. (**a**) quantitative reverse transcription-polymerase chain reaction (RT-PCR) analysis of the expression levels of overexpressed *BvM14-SAMS2* (OX1 and OX2) in *Arabidopsis* plants; (**b**) Structure of the *AtSAMS3* gene. The T-DNA insertional site was 756 bp upstream of the start codon, as indicated by a triangle. The primers used to identify the T-DNA insertion were marked with arrows; (**c**) PCR analysis of the T-DNA insertion in the *atsams3* mutant (KO); (**d**) RT-PCR analysis of the expression levels of *AtSAMS3* in *atsams3* mutant and wild type (WT); (**e**) Real-time PCR analysis of the expression levels of *AtSAMS3* in the *atsams3* mutant; (**f**) The concentration of *S*-adenosylmethionine (SAM) in 20-day old whole seedlings of wild type (WT), overexpressed *BvM14-SAMS2* (OX1 and OX2), *AtSAMS3* konckdown mutant (KO) and *BvM14-SAMS2* in mutant complementation seedlings (CO). Data are the means of three biological replicates (SD) and each replicate contains five seedlings. Different letters indicate significant difference at *p* < 0.05.

To determine the functions of *BvM14-SAMS2* under salt stress conditions, we created transgenic *Arabidopsis* plants that over-express the *BvM14-SAMS2* gene. Two homozygous T_3_ overexpressed *BvM14-SAMS2* transgenic lines (OX1 and OX2) were identified by RT-PCR analysis (Figure 3a). Furthermore, the T-DNA insertion mutant line of *atsam3* from *Arabidopsis* was identified (Figure 3b–d). The T-DNA was inserted in the upstream region of the *AtSAMS3* promoter (at 765 bp) (Figure 3b,c) and the expression level of *AtSAMS3* was significantly decreased in the *atsam3* mutant (Figure 3d,e). In addition, the construct overexpressing *BvM14-SAMS2* was also transformed into the *atsam3* mutant background and two homozygous T_3_ complementation lines (CO1 and CO2) were selected. Furthermore, the content of SAM was also detected in different *Arabidopsis* seedlings (Figure 3e). The *BvM14-SAMS2-*overexpressed transgenic lines showed higher levels of SAM than the wild type (WT), and *AtSAMS3* konckdown mutant (KO) line exhibited much lower SAM levels than WT. 

**Figure 4:**
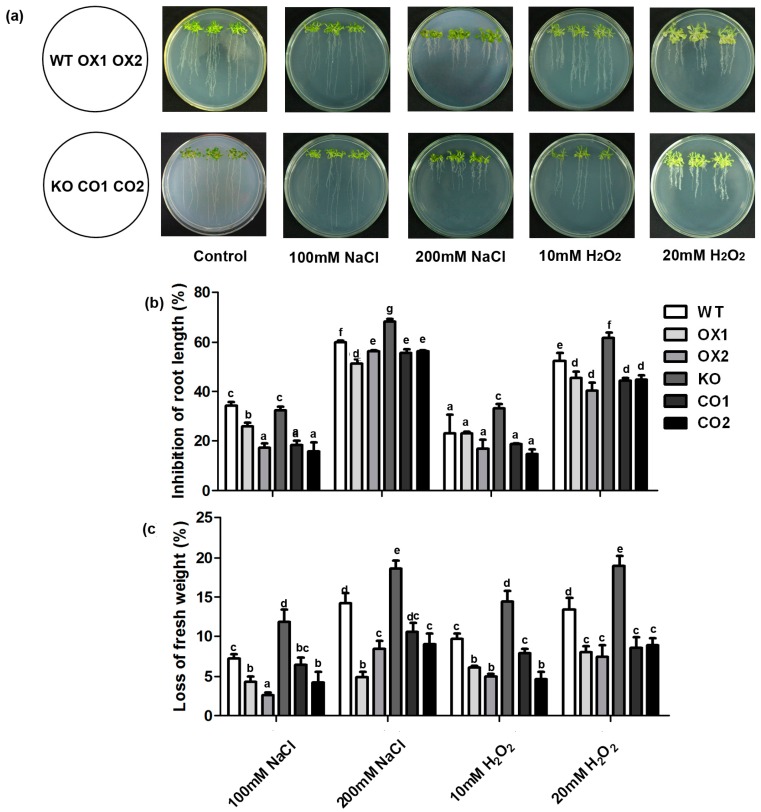
Analysis of salt and H_2_O_2_ tolerance in transgenic *Arabidopsis* plants in comparison with the wild type and *atsams3* mutant. (**a**) Phenotypes of wild type (WT), *BvM14-SAMS2 BvM14-SAMS2*-overexpressed seedlings in wild type *Arabidopsis* (OX), *atsams3* mutant (KO), and *BvM14-SAMS2* in mutant complementation seedlings (CO) under control and stress conditions. Photographs were taken 14 days after treatment. Inhibition of root length (**b**); and loss of fresh weight (**c**) were determined. Data are the means of three biological replicates (SD) and each replicate contains five seedlings. Different letters indicate significant difference at *p* < 0.05.

**Figure 5:**
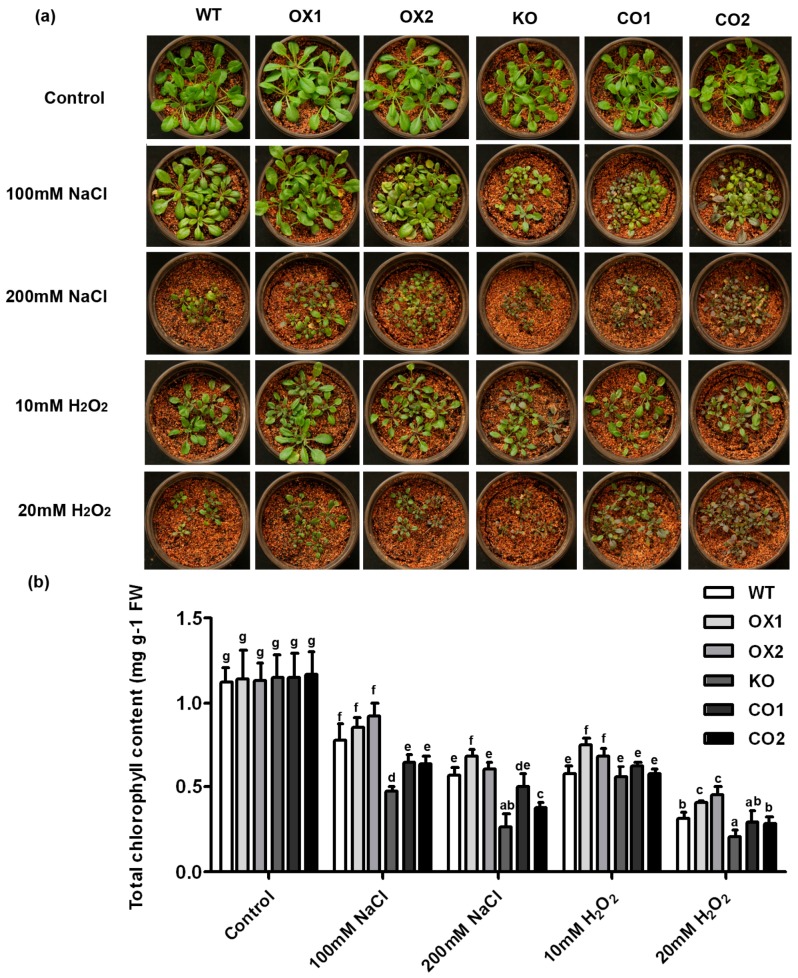
Analysis of salt and H_2_O_2_ tolerance in transgenic *Arabidopsis* plants in comparison with the wild type and *atsams3* mutant in soil. (**a**) Phenotypes of wild type (WT), *BvM14-SAMS2 BvM14-SAMS2*-overexpressed seedlings in wild type *Arabidopsis* (OX), *atsams3* mutant (KO), and *BvM14-SAMS2* in mutant complementation seedlings (CO) under conditions of control and stress in soil. Photographs were taken 7 days after treatment; Total chlorophyll levels (**b**) were determined. Data are the means of three biological replicates (SD) and each replicate contains five seedlings. Different letters indicate significant difference at *p* < 0.05.

The transgenic *Arabidopsis* lines were analyzed in salt stress tolerance on Murashige and Skoog (MS) plates. Overexpression of *BvM14-SAMS2* in *Arabidopsis* did not show growth inhibition under normal conditions. However, obvious wilting was found in all the seedlings under salt stress, the OX lines exhibited lower reduction in fresh weight and root length compared with the wild type or the *atsams3* mutant under salt stress (Figure 4). Similar phenotype was also identified under H_2_O_2_ stress (Figure 4). Furthermore, KO lines are more sensitive to salt and H_2_O_2_ stresses than the *atsams3* complementation seedlings (CO1/CO2) or wild type. In order to confirm the above results, the transgenic seedlings were treated with salt and H_2_O_2_ stress in soil. After 7 days of the 100 mM salt or 20 mM H_2_O_2_ stress treatments, plants overexpressing *BvM14-SAMS2* showed lower levels of wilting than wild type (Figure 5a). In addition, salt and H_2_O_2_ stresses caused a decrease of total chlorophyll content in the control seedlings. Under salt stress conditions, no significant differences were found between wild type and *BvM14-SAMS2-*overexpressed lines. However, the contents of chlorophyll were higher in the overexpressed *BvM14-SAMS2* lines than wild type under H_2_O_2_ stress (Figure 5b). In addition, *atsams3* mutants showed higher reduction in chlorophyll contents than WT and CO lines under salt stress and H_2_O_2_ stress.

### 2.5. Overexpression of BvM14-SAMS2 Increased Antioxidative Activities in Arabidopsis

**Figure 6:**
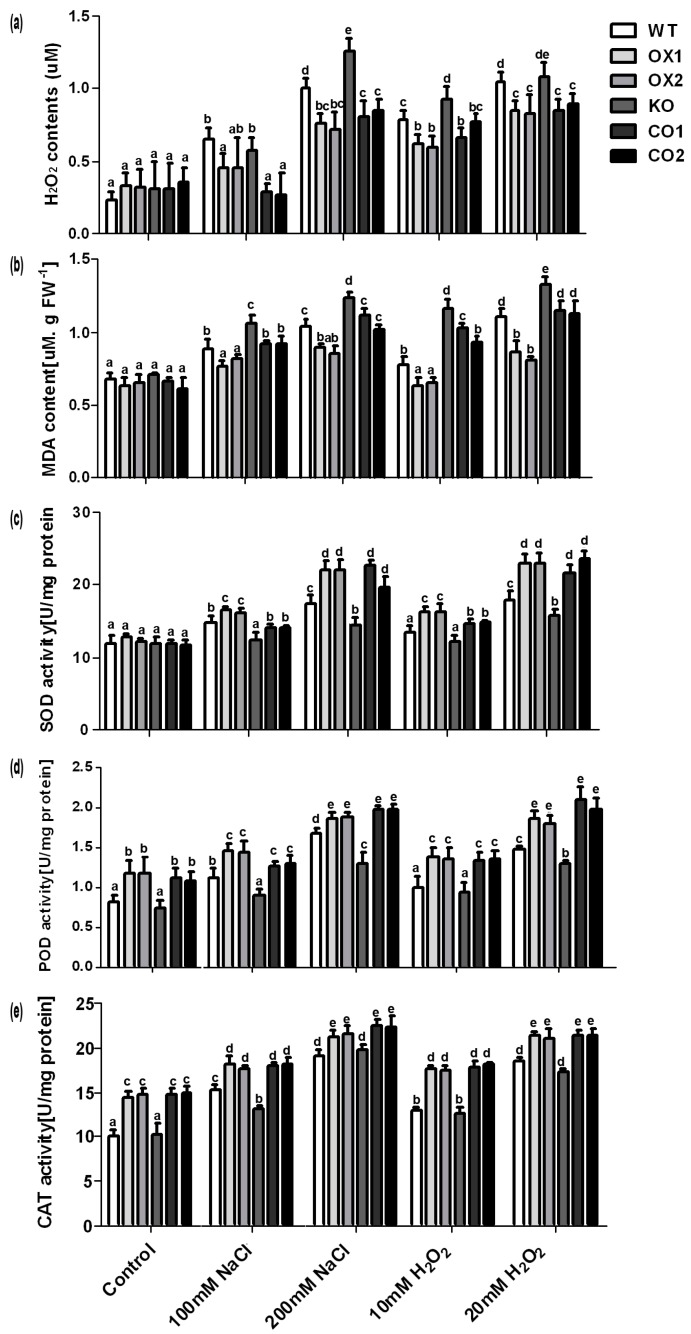
Effects of salt and H_2_O_2_ stresses on antioxidant system activity H_2_O_2_ content (**a**); malondialdehyde (MDA) content (**b**); and antioxidant enzyme activities (**c**–**e**) were measured in wild type (WT), transgenic *BvM14-SAMS2* wild type (WT), *BvM14-SAMS2*-overexpressed in wild type *Arabidopsis* (OX), *atsams3* mutant (KO) and transgenic *BvM14-SAMS2* in the mutant seedlings (CO) leaves. Data are the means of three biological replicates (SD) and each replicate contains five seedlings. Different letters indicate significant difference at *p* < 0.05.

Salt stress usually causes oxidation induced lipid membrane damage. The damage can be determined by lipid peroxidation, and the malondialdehyde (MDA) content reflects the level of lipid peroxidation. Under stress conditions, all genotype seedlings exhibited the increasing trend of H_2_O_2_ and MDA contents. However, KO lines accumulated higher contents of H_2_O_2_ and MDA than WT plants or CO lines (Figure 6a,b). Furthermore, overexpressed plants showed lower H_2_O_2_ and MDA contents than wild type plants under stress conditions (Figure 6a,b). These results demonstrated that oxidative damage was reduced in the overexpressed plants. In addition, we analyzed the activities of antioxidant enzymes to determine whether the antioxidant enzyme system was involved in reducing the oxidative damage. The activities of antioxidant enzymes including superoxide dismutase (SOD), catalase (CAT) and peroxidase (POD) exhibited higher activities in the OX lines than in WT under salt and H_2_O_2_ stresses (Figure 6c–e). Although stress conditions can induce the activities of these antioxidant enzymes in all the plants, the KO lines exhibited smaller extent of increasing activities than WT under salt and H_2_O_2_ stresses (Figure 6c–e). In addition, no differences in the activity of SOD were detected under the normal conditions between all the seedlings, except that the activities of CAT and POD in the overexpressed plants were higher than WT or KO line under the control conditions. 

### 2.6. Overexpression of BvM14-SAMS2 Greatly Influenced Polyamine Metabolism

**Figure 7:**
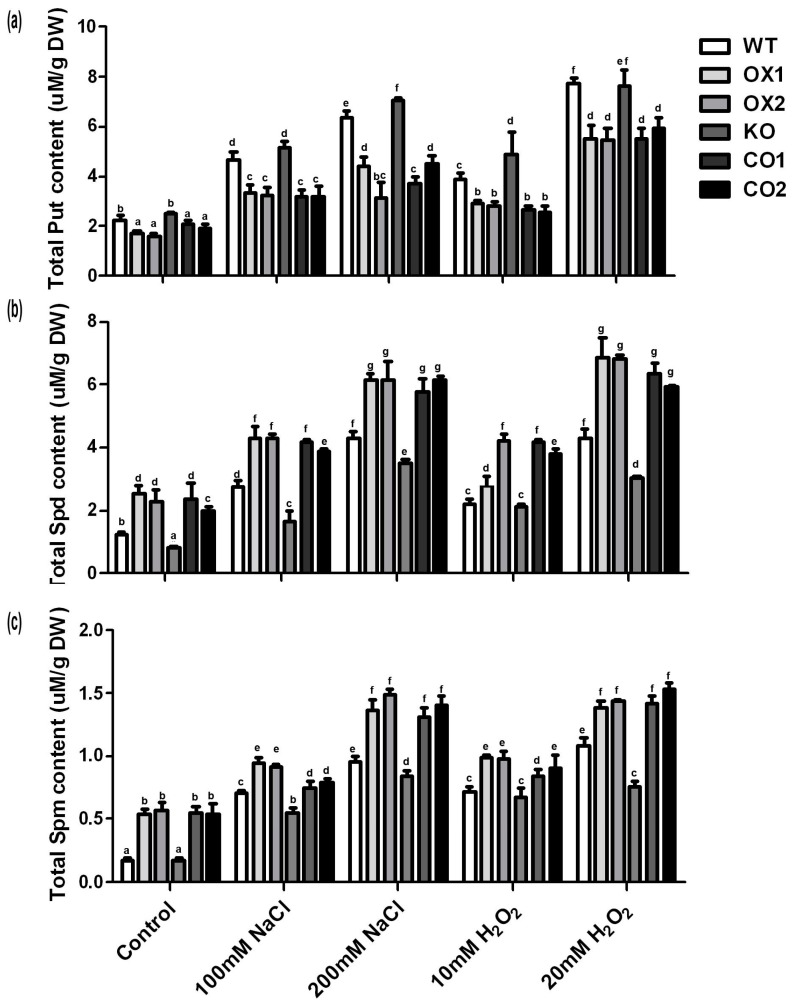
Effects of salt and H_2_O_2_ stresses on polyamines (PAs). Total putrescine (Put) content (**a**); total spermidine (Spd) content (**b**); total spermine (Spm) content (**c**) in wild type (WT), transgenic *BvM14-SAMS2* seedlings in wild type *Arabidopsis* (OX), *atsams3* mutant (KO) and *BvM14-SAMS2* in mutant complementation seedlings (CO) leaves. Data are the means of three biological replicates with standard deviation (SD), and each replicate contains five seedlings. Different letters indicate significant difference at *p* < 0.05.

Three types of polyamines, total putrescine (Put), spermidine (Spd) and spermine (Spm) were detected in the overexpressed transgenic plants and wild type. The OX lines showed lower Put concentration than the wild type under control, salt stress and H_2_O_2_ stress conditions (Figure 7a). However, under control and stress conditions, Spd and Spm increased in the OX lines (Figure 7b,c). These results showed that overexpressed *BvM14-SAMS2* greatly influenced polyamine metabolism. In addition, proline contents were analyzed (Figure S4) and the results showed that there were no differences between OX lines and wild type under the control condition. Under stress conditions, proline contents increased in both transgenic plants and wild type. However, proline contents were significantly higher in OX lines than in the wild type during salt and H_2_O_2_ stresses, indicating that the wild type plants experienced higher extent of cell damage than the transgenic plants under stress conditions.

## 3. Discussion

The *BvM14-SAMS2* characterized in this study was salt-stress induced in roots and leaves of the sugar beet M14 line. Our results are consistent with previous findings of the induction of *SAMS* expression by salt, cold, drought and H_2_O_2_ [28,29,30]. Furthermore, overexpression of *BvM14-SAMS2* in *Arabidopsis* led to significant increases in salt and H_2_O_2_ stress tolerance (Figure 4). Photosynthesis, as the most important and complex physiological process of plants, is severely affected by many abiotic stresses. It is reported that salt stress reduced the level of photosynthetic pigments. Usually, the level of photosynthetic pigment is thought to be a biochemical indicator for evaluating salinity tolerance in plants. The KO lines showed a decrease in the total chlorophyll content compared to WT under the stress conditions (Figure 5). Therefore, the decreased chlorophyll content clearly indicates the sensitivity of the KO line to salt and H_2_O_2_ stresses.

SAMS in *Arabidopsis* is encoded by four genes. *AtSAMS1* and *AtSAMS2* are expressed in most of plant tissues, including leaves, roots and flowers [31], whereas *AtSAMS4* is expressed predominantly in pollen [32]. Our data indicate that *AtSAM3* is also widely expressed in different plant tissues, and showed relatively high expression levels in roots. These results showed that *SAMS* is involved in many aspects of plant metabolism and development. *AtSAMS1* and *AtSAMS2* are most similar in sequence and expression patterns, and the double mutant *atsams1*/*atsams2* showed decreases in ethylene [31]. Furthermore, the *atsam4* mutant was impaired in pollen tube growth and reduced seed production [32]. Moreover, over-expression of the *AtSAMS1* in *Arabidopsis* leads to a dwarf phenotype [31]. In this study, overexpression of *BvM14-SAMS2* did not cause the dwarf phenotype. This result is similar to the overexpression of a potato *SAMS* in *Arabidopsis*, and the transgenic *Arabidopsis* lines exhibited high salt and drought stress tolerance [33]. Clearly, the *SAMS* gene family had function diversity and species specificity. In addition, *atsams3* plants decreased SAM content by more than 50%, as compared to the WT plants (Figure 3f). We speculate that different SAMSs catalyze SAM production at different seedling developmental stages, and AtSAMS3 may play a predominantly role in the 20-day old seedlings.

Reactive oxygen species (ROS) and MDA contents are proposed to be indicators of oxidative stress. Usually, reducing the use of absorption light energy caused by inhibition of calvin cycle enzyme under stress conditions will promote production of ROS [34]. Plants possess efficient enzymatic antioxidant defense systems to protect the cells from oxidative damage by scavenging ROS. For example, SOD dismutates superoxide radicals to H_2_O_2_, which is sequentially scavenged by CAT and POD [35]. Other reports showed that exogenous Spd enhanced chilling tolerance in tomato through enhancing the expression of SOD, POD, CAT and ascorbate peroxidase (APX), and their activities in tomato leaves [36]. Meanwhile, nitric oxide (NO) induced by Spd plays a crucial role in regulating these antioxidant enzymes. Similarly, in our study, the activities of CAT and POD were higher in the *BvM14-SAMS2* overexpression plants than WT or *atsams3* mutant under control and stress conditions. In addition, the low accumulation of H_2_O_2_ and MDA after H_2_O_2_ or salt stress in the *BvM14-SAMS2-*overexpressed plants was observed (Figure 6). These results showed that the OX lines possessed high antioxidant enzyme activities that help them to better cope with the stress conditions than the other plants. Furthermore, although the activity of SOD was not different between the genotypes in control conditions, the *BvM14-SAMS2-*overexpressed transgenic lines showed higher activity than WT or mutant under stress conditions. These results were similar to a previous study [37], where SOD was activated to reduce the ROS levels in *TaWRKY44* overexpression transgenic lines after drought and salt stresses [37]. Usually, SOD provides the first line of defense against ROS by catalyzing the dismutation of O_2_^−^ to oxygen and H_2_O_2_. Thus, our result indicated the antioxidant enzymes involved in first line of defense in *BvM14-SAMS2* overexpression plants function more effectively than the WT or KO line.

Recently, several studies have shown the interplay between PAs and signal molecules (e.g., abscisic acid (ABA) and ethylene) under abiotic stresses [38]. It is reported that exogenous Put significantly increased the ABA content in tomato [38]. Furthermore, the expression levels of genes related to ethylene biosynthesis and ABA response were up-regulated in *SbSAMS*-overexpressing Arabidopsis lines, and the transgenic *Arabidopsis* plants exhibited higher salt and drought stress tolerance than control plants [33]. Taken together, it is speculated that, under salt or H_2_O_2_ stresses, the increased Spd and Spm contents due to the overexpressed *BvM14-SAMS2* may affect the stress-related genes including the ethylene and ABA genes responsible for abiotic stress response and tolerance.

In the process of PA synthesis, SAM is converted to decarboxylated *S*-adenosylmethionine (dcSAM), which can supply an aminopropyl group donor for the synthesis of PA. PA is well-known to play an important role in regulating plant adaptation to abiotic stresses [19]. PAs have been reported to scavenge ROS in plants [39] and are thought to be membrane protectors [40]. The contents of the diamine (Put) and triamine (Spd and Spm) were analyzed here. The levels of these common PAs were induced under salt stress conditions in the wild type plants. However, the level of Put was much lower in the *BvM14-SAMS2* overexpression lines under both control and salt stress conditions, while the contents of Spd and Spm were much higher in the *BvM14-SAMS2* overexpression lines, leading to a high ratio of (Spd + Spm)/Put. Other reports showed that Spd and Spm play a vital role in maintaining the thylakoid membrane integrity. Nevertheless, Put may be involved in depolarization of the membrane [21,41]. Therefore, a high ratio of (Spd + Spm)/Put in the *BvM14-SAMS2* overexpression lines may be an important factor for the salt stress tolerance in the M14 plants. This trend was observed not only in the sugar beet M14 under control and salt stress conditions, but also in tomato plants under high level alkali stress [21]. It is reported that arginine decarboxylase (ADC) and omithine decarboxylase (ODC), two key enzymes in Put biosynthesis, exhibited much higher activities in *SlSAMS* overexpression lines under alkali stress, while they did not cause higher accumulation of Put. They speculated that up-regulation of *SlSAMDC* and *SlSPDS* may be the main cause of this phenomenon. High levels of Put were quickly converted to Spm and Spd by *SlSAMDC* and *SlSPDS.* Thus, it may be concluded that salt or H_2_O_2_ stress tolerance induced by overexpressing the *BvM14-SAMS2* might be involved in increasing the SAM levels for generating Put (Figure 3f), and enhanced conversion of Put to Spd and Spm has been suggested to play important roles in plant tolerance to stress conditions.

## 4. Materials and Methods 

### 4.1. Plant Materials and Growth Conditions

Seeds of monosomic addition line M14 were sown in vermiculite and watered daily. After one week, seedlings were transferred to hydroponic containers with Hoagland solution. Seedlings were cultivated in a greenhouse at Heilongjiang University with a 12 h/12 h light/dark, a 450 μmol·m^−2^·s^−1^ light intensity, a 24 °C/20 °C day/night temperature, and a relative humidity of 70%. *Arabidopsis* growth and treatment were conducted as previously described [27].

### 4.2. Construction of a Subtractive cDNA Library

Total RNAs were extracted using TRIzol reagent (Invitrogen, Carlsbad, CA, USA) from the salt stress and the control samples and mRNA was selected by the Oligotex mRNA Kit (Qiagen, Los Angeles, CA, USA). A subtractive cDNA library was generated by the method of our previous report using the salt-treated roots and leaves as the tester and the control roots and leaves as the driver [6]. 

### 4.3. Molecular Cloning of BvM14-SAMS2 Gene and Sequence Analysis

An EST named as Me-359 matching to *BvM14-SAMS2* was identified. The 0.4-kb cDNA sequence was amplified by the method of reverse transcription RT-PCR using two primers: 5′-GTCTGATGATGTGGGTCTTGATGCT-3′ (sense primer) and 5′-GAGTCTTACCATCAGGTCTC AGCCA-3′ (antisense primer). Furthermore, the full length of *BvM14-SAMS2* was acquired by the method of Smart-RACE (Clontech, MountainView, CA, USA). Phylogenetic tree of *BvM14-SAMS* genes was made using the ClustalX program combined with MEGA 4 software [42]. 

### 4.4. Real-Time Quantitative PCR

First-strand cDNA was generated from 0.5 µg of total RNA, using ReverTra Ace reverse transcriptase (Toyobo, Tokyo, Japan). The *BvM14-SAMS2* specific primers (5′-GTCTGATGATGTGGGTCTTGATGCT-3′ and 5′-GAGTCTTACCATCAGGTCTCAGCCA-3′) were used for real-time quantitative RT-PCR. In order to verify specific of *BvM14-SAMS2* primer, we conducted an analysis using Primer-BLAST tool in sugar beet *(Beta vulgaris)* genome. This pair of primers was specifically matched on *SAMS2* in sugar beet genome (Figure S5). Furthermore, randomly-selected 10 positive clones from PCR products from this pair of primers were sequenced, and only *BvM14-SAMS2* sequences can be identified. The 18S rRNA gene (primers: 5′-CCCCAATGGATCCTCGTTA-3′ and 5′-TGACGGAGAATTAGGGTTCG-3′) was used as an internal control. The reaction system contained aliquots of cDNA (1/20) of 1 μL, 150 nM each for forward and reverse primers and 5 μL SYBR Premix Ex Taq (Takara, Kusatsu Shiga, Japan) in total for the 10 µL PCR mixture. The cycle threshold (CT) for internal control should be between 15 and 20. A negative control without cDNA template was always included. The experiment was conducted by a LightCycler480 (Roche, Penzberg, Germany) instrument according to the manufacturer’s instructions. Triplicate quantitative assays were performed on each cDNA sample.

### 4.5. Isolation of T-DNA Insertion Mutants in AtSAMS3

*Arabidopsis* T-DNA insertion lines were identified for *AtSAMS3* mutation by PCR. Specific primers for the left and right borders of the T-DNA and for *AtSAMS3* (F1: 5′-CTAGATCTCATTGTCTGAACACAGTT-3′, R1: 5′-CGTACAGGAACCATGGCTCCGCTTT-3′, T1: 5′-TAGCATCTGAATTTCATAACCAATCTCGATACAC-3′) were used to identify mutant lines.

### 4.6. Constitutive Expression of BvM14-SAMS2 in Arabidopsis and Stress Tolerance Analysis 

The coding region of the *BvM14-SAMS2* gene was amplified using primers (5′-GCG AGATCTTCTCTCACTCTCTTCGTCCAGG-3′ and 5′-GCGACTAGTGCATTACGCAGGGATTTTC-3′). Then, it was ligated into the PMD18-T vector. It was cut with *Bgl*II and *Spe*I and ligated into the *Bgl*II and *Spe*I sites of a binary vector pCAMBIA1305.1 under the control of CaMV35S promoter. The construct was introduced into the *Agrobacterium tumefaciens* EHA105. *Arabidopsis* was transformed by the floral dip method [27]. The expression of *BvM14-SAMS2* in different homozygous lines selected at the concentration of 50 μg/mL Kanamycin was confirmed by the method of RT-PCR. The content of chlorophyll was measured in accordance with a previous report [43].

### 4.7. Determination of Antioxidant Enzyme Activities, Lipid Peroxidation, H_2_O_2_ Concentration, SAM Concentration and Polyamine Metabolism

Measurement of concentration H_2_O_2_ was performed as previously described [21]. For the antioxidant enzyme assays, SOD activity was measured by analyzing its ability to inhibit the photochemical reduction of nitrobluetetrazolium following the method of Guo et al. [22]. CAT and POD activities were analyzed according to a previous report [21]. SAM concentration was determined following the method of Roeder et al. [44]. PA contents were assayed in accordance with a method described by Hu et al. [20]. Lipid peroxidation was estimated by determining the malondialdehyde (MDA) content in the leaves. For MDA extraction, fresh leaf samples (0.5 g) were homogenized with 0.1% trichloroacetic acid (TCA). The homogenate was then centrifuged at 15,000× *g* for 10 min. An aliquot (1 mL) of the supernatant was mixed to 4 mL of 20% TCA prepared in 0.5% thiobarbituric acid (TBA) and incubated at 90 °C for 30 min in a shaking water bath. The reaction was stopped in ice bath. The samples were then centrifuged at 10,000× *g* for 5 min, and the absorbance of the supernatant was measured at 532 and 600 nm. 

### 4.8. Statistical Analysis

All the data were subjected to analysis of variance according to the model for completely randomized design using an SPSS program (SPSS Inc., Chicago, IL, USA). Differences among means of treatments and plant lines were evaluated by the Duncan’s test at 0.05 probability level.

## 5. Conclusions

In summary, the expression of *BvM14-SAMS2* is significantly induced by salt treatment. Our study showed that overexpression of *BvM14-SAMS2* significantly conferred salt and H_2_O_2_ stress tolerance in transgenic *Arabidopsis* plants. The functions of *BvM14-SAMS2* are mainly accomplished through increased accumulation of Spd, Spm and activities of the antioxidant system, which are involved in scavenging ROS under stress conditions.

## Figures and Tables

**Table 1:** cDNA clones isolated from a subtractive hybridization library of salt stressed sugar beet M14 roots (R) and leaves (L).

Unigene Number	Length (bp)	Annotation	Score	*E*-Value
L1	354	Glutathione *S*-transferase	159	6 × 10^−33^
L2	231	Late embryogenesis abundant protein	73	1 × 10^−5^
L3	174	Chloroplastic chlorophyll a-b binding 8	154	2 × 10^−27^
L4	150	ATP-binding cassette transporter C family member 14	70	3 × 10^−11^
L5	392	Ca^2+^ transporting ATPase	260	4 × 10^−78^
L6	237	MYB (v-myb avian myeloblastosis viral oncogene homolog) transcription factor	52	2 × 10^−7^
L7	359	Salt-induced hydrophilic protein	70.3	2 × 10^−9^
L8	467	Aldehyde dehydrogenase family 7 A1	119	1 × 10^−32^
L9	233	Cysteine proteinase inhibitor	134	1 × 10^−8^
L10	221	Aldehyde dehydrogenase	123	3 × 10^−13^
L11	274	*S*-adenosylmethionine decarboxylase	155	1 × 10^−36^
L12	148	Pyruvate kinase family protein	89	4 × 10^−18^
L13	274	Cysteine protease	63	1 × 10^−7^
L14	297	Short-chain dehydrogenase	73	7 × 10^−11^
L15	246	Carboxyl-terminal-processing protease	132	1 × 10^−30^
L16	301	MYB transcription factor	94	2 × 10^−7^
L17	144	Short-chain dehydrogenases/reductases family 7C	198	5 × 10^−8^
L18	326	Vacuole ATPase subunit A	144	3 × 10^−66^
L19	528	Putative mitochondrial carrier protein	163	5 × 10^−77^
L20	416	Nitrite reductase	254	4 × 10^−66^
L21	512	Lipid transfer protein	184	4 × 10^−45^
L22	314	*S*-adenosylmethionine synthase 2	239	1 × 10^−61^
L23	763	Phosphoglycerate kinase	407	8 × 10^−112^
L24	236	Cellulose synthase-like protein E6	71.2	5 × 10^−11^
L25	450	High-mobility group B6 transcription factor	69	3 × 10^−2^
L26	544	Heat- and acid-stable phosphoprotein	95	3 × 10^−16^
L27	236	Thioredoxin domain 2	68	6 × 10^−31^
L28	437	Hypothetical protein	109	5 × 10^−4^
L29	197	DNA replication licensing factor minichromosome maintenance 4 (MCM4)	88	2 × 10^−2^
R1	245	Thioredoxin domain 1	96	3 × 10^−13^
R2	364	S-adenosylmethionine synthase 2	194	1 × 10^−22^
R3	208	WD (Trp-Asp) repeat phosphoinositide-interacting	174	6 × 10^−30^
R4	447	AAA-type ATPase family protein	143	4 × 10^−12^
R5	182	Putative senescence-associated protein	121	6 × 10^−2^
R6	394	*S*-adenosylmethionine synthase 2	74	3 × 10^−7^
R7	113	60S ribosomal protein L19-3	863	4 × 10^−14^

## Supplementary Materials

Supplementary materials can be found at www.mdpi.com/1422-0067/18/4/847/s1.
