# Supplementary material for: Overexpression of S-Adenosyl-l-Methionine Synthetase 2 from Sugar Beet M14 Increased Arabidopsis Tolerance to Salt and Oxidative Stress
Source: Int J Mol Sci. 2017 Apr 18;18(4):847. doi: 10.3390/ijms18040847 (PMC5412431; doi:10.3390/ijms18040847)
Supplement: Supplementary file 1 [file ijms-18-00847-s001.pdf]

Supplementary Materials: Overexpression of *S*-Adenosyl-L-Methionine Synthetase 2 from Sugar beet M14 Increased *Arabidopsis* Tolerance to Salt and Oxidative Stress

Chunquan Ma, Yuguang Wang, Dan Gu, Jingdong Nan, Sixue Chen and Haiying Li

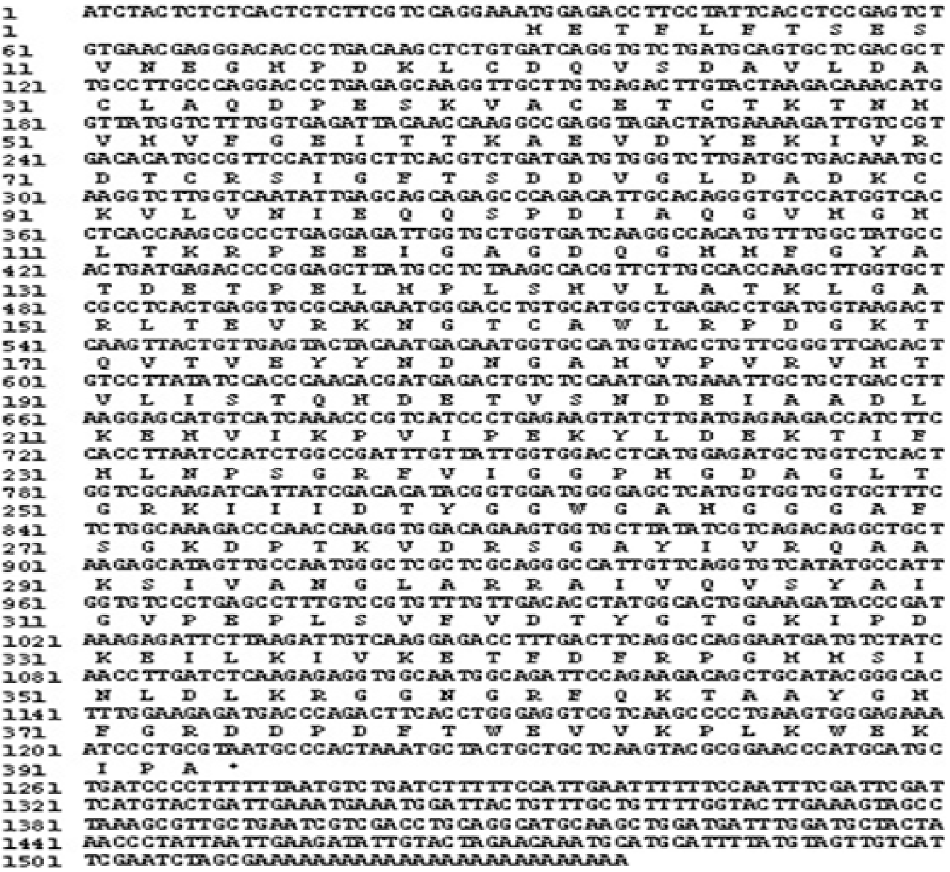

Figure S1. Sequence analysis of a cDNA encoding a *BvM14-SAMS2* isolated from the monosomic addition line M14 roots. Nucleotide and deduced amino acid sequence of *BvM14-SAMS2*.

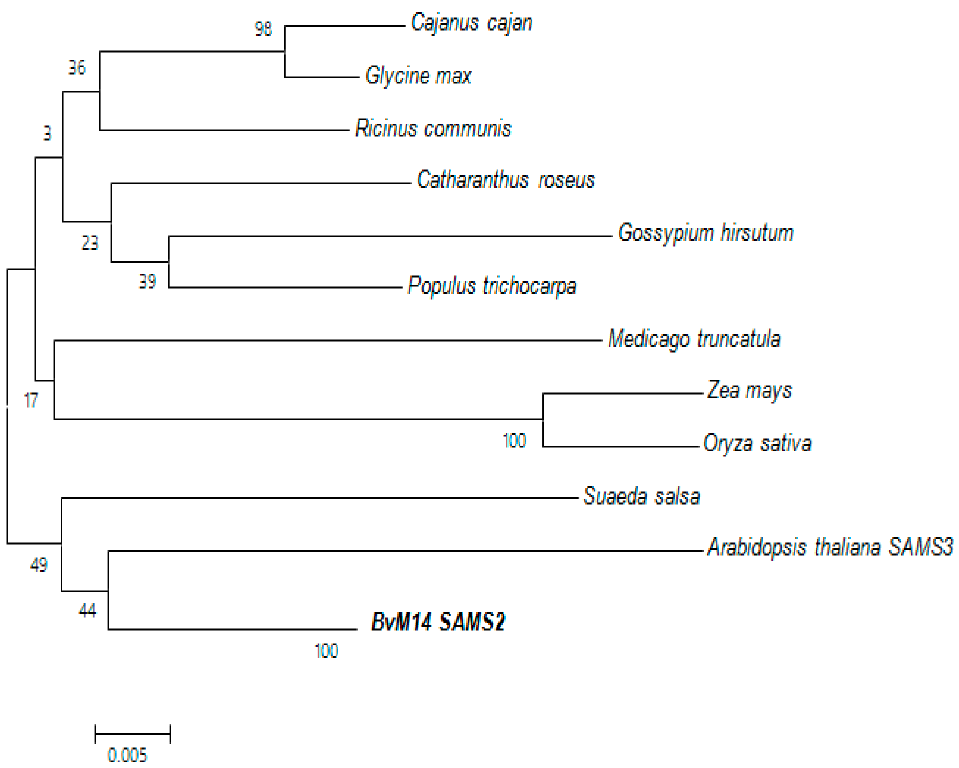

Figure S2. Phylogenetic tree of *BvM14-SAMS2* and *SAMS* gene members. The name of the gene (*BvM14-SAM2*) isolated in this study is in bold.

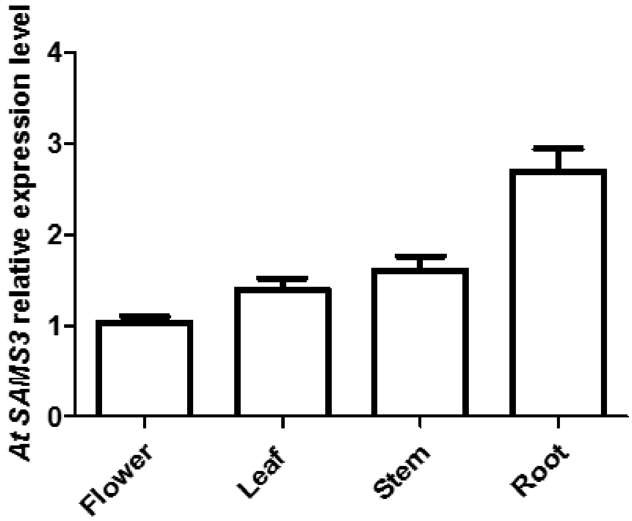

Figure S3. Tissue specific expression of *AtSAMS3* gene in *Arabidopsis* plants

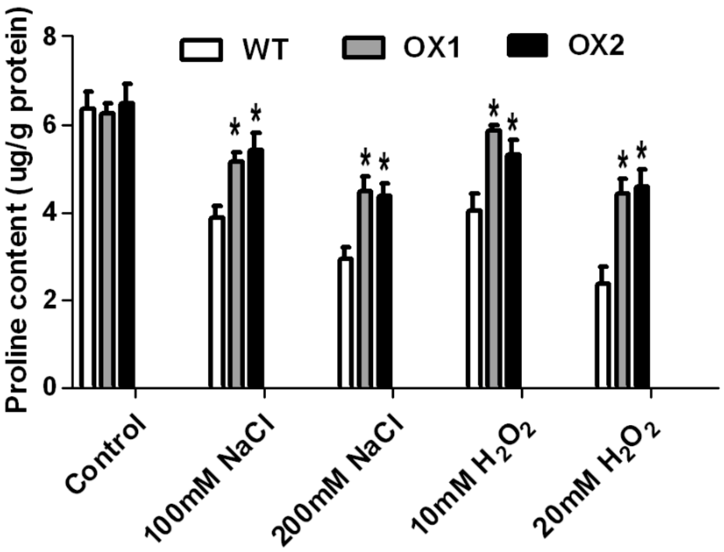

Figure S4. Effects of salt and H_2O_2 stress on proline content in wild type (WT) and *BvM14-SAMS2*-overexpressed seedlings in wild type Arabidopsis (OX).

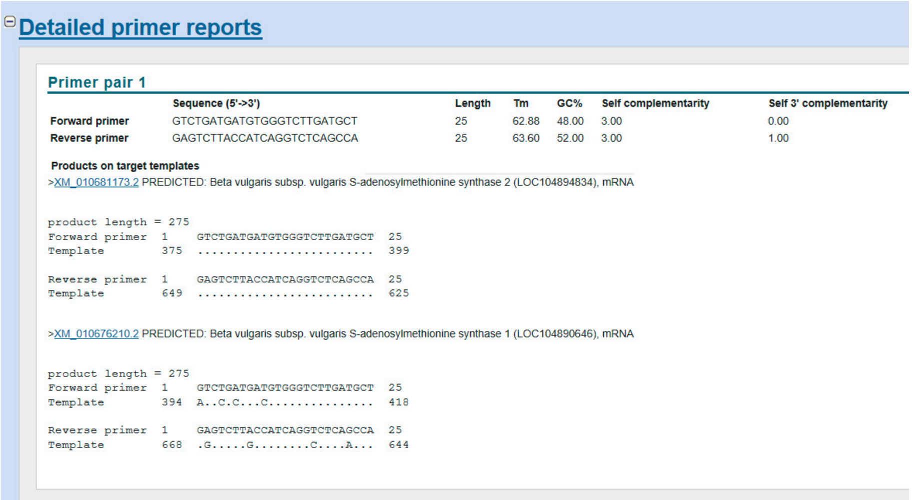

Detailed primer reports

Primer pair 1

	Sequence (5'->3')	Length	Tm	GC%	Self complementarity	Self 3' complementarity
Forward primer	GTCTGATGATGTGGGTCTTGATGCT	25	62.88	48.00	3.00	0.00
Reverse primer	GAGTCTTACCATCAGGTCTCAGCCA	25	63.60	52.00	3.00	1.00

Products on target templates

>XM_010681173.2 PREDICTED: Beta vulgaris subsp. vulgaris S-adenosylmethionine synthase 2 (LOC104894834), mRNA

```
product length = 275
Forward primer  1    GTCTGATGATGTGGGTCTTGATGCT  25
Template        375  .........................  399

Reverse primer  1    GAGTCTTACCATCAGGTCTCAGCCA  25
Template        649  .........................  625
```

>XM_010676210.2 PREDICTED: Beta vulgaris subsp. vulgaris S-adenosylmethionine synthase 1 (LOC104890646), mRNA

```
product length = 275
Forward primer  1    GTCTGATGATGTGGGTCTTGATGCT  25
Template        394  A..C.C...C...............  418

Reverse primer  1    GAGTCTTACCATCAGGTCTCAGCCA  25
Template        668  .G.....G........C....A...  644
```

Figure S5. Primer-BLAST result for *BvM14- SAMS2* in sugar beet genome.
